# New Avenues for Nanoparticle-Related Therapies

**DOI:** 10.1186/s11671-018-2548-8

**Published:** 2018-05-08

**Authors:** Michael Zhao, Mingyao Liu

**Affiliations:** 1 Latner Thoracic Surgery Research Laboratories, Toronto General Hospital Research Institute, University Health Network, 101 College Street, Room: TMDT2-814, Toronto, Ontario M5G 1L7 Canada; 2 Institute of Medical Science, University of Toronto, Toronto, Canada; 3 Department of Surgery, University of Toronto, Toronto, Canada; 4 Department of Medicine, University of Toronto, Toronto, Canada; 5 Department of Physiology, University of Toronto, Toronto, Canada

**Keywords:** Nanodrug delivery, Systemic diseases, Local delivery, Ex vivo lung perfusion, Translational research

## Abstract

Development of nanoparticle-based drug delivery systems has been attempted for the treatment of cancer over the past decade. The enhanced permeability and retention (EPR) effect is the major mechanism to passively deliver nanodrugs to tumor tissue. However, a recent systematic review demonstrated limited success of these studies, with the clearance of nanoparticles by the mononuclear phagocytic system (MPS) being a major hurdle. Herein, we propose that nanotechnologists should reconsider their research focuses, aiming for therapeutic targets other than cancer. Treatments for diseases that do not (or less) rely on EPR should be considered, such as active targeting or MPS evasion systems. For example, systemic delivery of drugs through intravenous injection can be used to treat sepsis, multi-organ failure, metabolic disorders, blood diseases, immune and autoimmune diseases, etc. Local delivery of nanodrugs to organs such as the lung, rectum, or bladder may enhance the local drug concentration with less clearance via MPS. In transplant settings, ex vivo organ perfusion provides a new route to repair injury of isolated organs in the absence of MPS. Based on a similar concept, chemotherapy with in vivo lung perfusion techniques and other isolated organ perfusion provides opportunities for cancer therapy.

## Background

Over the past decade, the explosion of nanoparticle-related drug delivery research has outpaced that of gene therapy and human embryonic stem cell-based therapy research. As with gene therapy and embryonic stem cell research, the main focus of nanoparticle research is a cure for cancer and optimism for other diseases. The enhanced permeability and retention (EPR) effect is considered the major mechanism for nanoparticle-related therapy in cancer [1]. However, a recent study conducted by Wilhelm et al. serves as a sharp rebuke to the effectiveness of this mechanism. This meta-analysis examines nanoparticle research over the past decade and reveals that only a median of 0.7% of the injected doses of nanoparticles passively reached target tumors, a percentage too low to have significant pharmacological effect after translating to a human equivalent dosage [2]. Indeed, this is reflected by the relatively few nanoparticle therapies approved for market by the Food and Drug Administration [3]. Moreover, well-known approved nanotherapies, such as Abraxane and Doxil, do not provide improved therapeutic index or diagnostics. Rather, they have improved toxicological profile over their “naked” drug form [2, 4]. The dosimetry analysis of Wilhelm et al. demonstrates that translation of nanoparticle therapy for tumors requires more understanding of basic nanoparticle interactions, and a 30-year research plan proposed [2] indicates that we need to re-think the directions of nanoparticle-related therapy research.

**Fig. 1:**
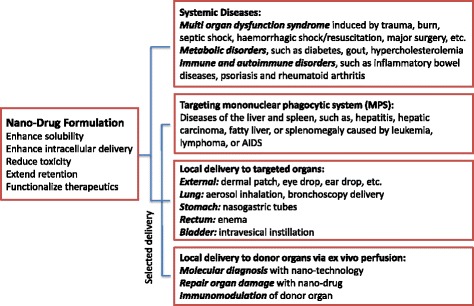
New avenues for nanoparticle-related therapies. Potential application of nanodrug formulations for clinical situations less relies on passive enhanced permeability and retention (EPR) effects

In addition to long-term plans aimed at systematically exploring the mechanisms and methodologies that may improve foundational nanotechnology understanding, for example, active targeting strategies with peptides, antibodies, or other types of ligands that specifically target to certain types of cancer cells, new avenues to translate potential nanoparticle-related therapies to clinical practice are required. We should think “outside the box” to convert the limitations of nanoparticle delivery to therapeutic advantage; aim for therapeutic targets other than cancer; develop systemic delivery of therapeutics for sepsis, organ failure, metabolic disorders, blood diseases, and immune and autoimmune diseases; and develop local delivery of nanoparticle therapies to target organs either in vivo (inside the body) or ex vivo (outside of the body) (Fig. 1).

## Converting the Limitations of Nanoparticle Delivery to Therapeutic Advantages

The primary cause of the ineffective delivery, and thus translation of nanotherapies, is the capture of the vast majority of the nanoparticles by the mononuclear phagocytic system (MPS). The MPS is composed of monocytes and macrophages primarily located in the liver, spleen, and lung, which selectively capture nanometer-sized particles to regulate homeostasis and microbial immunity. Nanoparticles are efficiently sequestered to these cell populations and accumulate in these organs in varying proportions dependent on nanoparticle size, shape, and surface modification. Attempts have been made to overcome these challenges, for instance, stabilizing particles sterically rather than electrostatically [5], conjugating “self” component(s) to the nanoparticle surface [6], and coating nanoparticles with cell membranes extracted from red blood cells or leukocytes [7]. Despite these developments, there remains a lack of understanding concerning the precise chemical interactions between nanoparticles and cells and organ architecture of the MPS.

However, if we reframe our therapeutic objectives to the diseases that affect the major organs of the MPS, such as the liver, spleen and lungs, the accumulation of nanoparticles in these organs may enhance delivery and therapeutic efficacy of nanoparticle-conjugated drugs. Therapeutic treatments of liver inflammation and fibrosis by nanomedicine with cell-specific targeting strategies have been proposed [8]. Moreover, for drugs that may not need to enter into cells or drugs that have broad effects on multiple cell types, enriched tissue concentrations could be beneficial. Therefore, other diseases of the liver and spleen, such as hepatitis, hepatic carcinoma, fatty liver, or splenomegaly caused by leukemia, lymphoma, or AIDS, could also be targets of nanomedicine. An attractive group of diseases affecting the cells of the MPS are the genetically inherited and severe lysosomal storage diseases and glycogen storage diseases, which may be attractive targets of pharmaceutical companies, as these are rare diseases and treatments may gain lucrative orphan drug statuses.

## Changing the Targets: Application of Nanoparticle Therapy for Systemic Diseases

A systematic review on nanoparticle clinical trials demonstrates that the majority of biomedical nanoparticle research and conceptual thinking takes place within the context of treating tumors [9]. Effective treatment of tumors requires long-term retention of drugs carried by nanoparticles in tumor tissue, which is severely hindered by the MPS. Instead of focusing on cancer, nanotechnologists should collaborate with clinicians to develop new therapeutics that target systemic diseases, which do not rely on EPR.

Many infectious diseases, viral, bacterial, and fungal infections, are systemic. Even though antibiotic, anti-viral, and anti-fungal drugs are available, systemic inflammatory responses, septic shock, and multi-organ dysfunction syndrome are life-threatening. Multi-organ dysfunction syndrome can also be induced by trauma, burn, hemorrhagic shock/resuscitation, major surgery, etc. Other than life support, there is no specific clinical therapy. Experimentally, many drugs have been found effective in animal models; however, to make them clinically available, nanotechnology is required to improve drug delivery. Attempts to make intravenously injectable formulas for hydrophobic drugs have been undertaken [10, 11]. Gold nanoparticles have been used as carriers to deliver peptide drugs that target Toll-like receptors or block intracellular signal transduction pathways of excessive inflammatory responses [12, 13]. Nanoparticle-based therapy could open a new avenue in this line of research.

Other systemic diseases may also be benefited by nanoparticle-related therapies. Nanoparticle-based anti-diabetic drug delivery has been developed [14]. Functionalized nanoparticles have been considered in treatments for gout [15]. Other metabolic disorders, such as hypercholesterolemia, may also be benefited by nanomedicine.

In hematology, by blocking drug efflux, nanotechnology may counter multiple drug resistance in leukemia [16]; gold nanoparticle has been used as a nanocarrier for anti-leukemic drugs [17]. Nanoparticles can be engineered to be pro-coagulant or to carry coagulation-initiating factors to treat disorders in blood coagulation. They can also be designed to be anticoagulant or to carry anticoagulant drugs [18, 19]. Nanoparticle-based thrombolytic agents may improve clot removal [16].

Nanoparticles can also be used for treatments of immune and autoimmune disorders. Capture of nanoparticles by MPS cells can be used as a targeting strategy for innate immune cells, such as macrophages, dendritic cells, and neutrophils, to treat inflammatory diseases and autoimmune disorders, such as inflammatory bowel diseases, psoriasis, and rheumatoid arthritis [20, 21]. Allergen-specific immunotherapy is a cause-oriented therapy for allergic asthma and rhinoconjunctivitis. Encapsulation of allergens or DNA vaccines into nanostructures may reduce their degradation, enhance local concentration and targeted delivery, and prevent recognition of allergens by antibodies [22]. Synthetic nanoparticles play a significant role in vaccine design and development [23].

For many systemic diseases, multiple organ systems and multiple cell types are involved. For example, excessive inflammatory responses and different types of cell death are seen in many systemic diseases. Using broad therapeutics that has beneficial effects in multiple cell types could be advantageous. In these contexts, nanoparticles are taken advantage of as delivery platform to enhance the solubility of hydrophobic drugs, allow delivery of peptide drugs into cells, reduce the toxicity of drugs, and extend the retention of medication. Moreover, nanoparticles may be functionalized to enhance the therapeutic effects [21].

## Local Delivery of Nanoparticle Therapies to Targeted Organs In Vivo

The concept of drug delivery to specific areas of the body is not a new idea: intraocular and ear drops, dermal patches, and inhalation of aerosolized drugs are all used to achieve higher drug concentration in their treatment areas. However, how to apply nanotechnology to enhance local delivery should be given more consideration. Nanotechnology could be used to further modify and enhance the local drug delivery in vivo [16]. For example, a hydrophobic compound, PP2 (Src protein tyrosine kinase inhibitor) has been incorporated in a nanoformulation using self-assembly peptides and amino acids to enhance intra-tracheal delivery and reduce acute lung injury [10].

In addition to the lung, the stomach, rectum, and bladder are relatively easy targets for local drug delivery. Gastric access via nasogastric tubes or gastrostomy tubes provides route for nutrition support and drug delivery [24]. Enema has been used for topical administration of medication into the rectum, for the treatment of inflammatory bowel diseases, ulcerative colitis, and other diseases, which avoids having the medication passing through the whole gastrointestinal tract [25]. The intravesical instillation of drugs has been used to treat superficial bladder cancer [26], painful bladder syndrome and recurrent urinary tract infection [27], and other diseases. Nanoparticle-based drugs can be delivered through these techniques.

With the development of minimally invasive surgery, more internal organs can be reached for local therapy. For example, the ability of nanoparticles to permeate into and/or retain in the inflamed joint after intra-articular administration has been beneficial in improving rheumatoid arthritis therapy while reducing systemic exposure to potentially toxic drugs [28]. Nanoparticles can stabilize and carry biomaterials across the round window membrane into the inner ear, which has been developed for the treatment of sensorineural hearing loss [29].

## Local Delivery of Nanoparticle Therapies to Targeted Organs Ex Vivo

In lung transplantation, the development of the ex vivo lung perfusion (EVLP) system provides an opportunity to assess the function of donor lungs. Donor lungs preserved at low temperature are gradually warmed to body temperature, ventilated, and perfused for functional assessment. This has increased the number of lung transplants with satisfactory quality [30]. Moreover, the EVLP technique provides a platform for organ repair [31]. Multiple therapeutics, including drugs, anti-inflammatory interleukin-10 gene therapy, antibiotics, and mesenchyme stromal cells, have been tested for efficacy in EVLP [32–35]. EVLP is ideal for the effective delivery of nanoparticle-related therapy. In the isolated lung, the dosage of therapeutics can be significantly reduced. In the absence of the liver, spleen, and kidney, the loss of nanoparticles through these organs is eliminated. Using EVLP as a platform, therapeutic effects can be tested without risk of patients. Only donor lungs that meet the clinical criteria will be used for transplantation. Moreover, pilot studies for ex vivo organ perfusion are currently in development for kidney [36], heart [37], and liver [38] transplantation. Some initial evidence for the possible success of such strategies comes in a recent study, where a small interfering RNA nanoparticle was delivered to human arterial allografts during ex vivo perfusion and successfully knocked-down MHC class II when transplanted to immunodeficient mouse hosts [39]. Furthermore, ex vivo organ perfusion can be used as a model to study how nanomaterials are processed within specific organs in a simplified environment. This will help us understand the pharmacodynamics of nanoparticle-based therapeutics in vivo and further improve drug delivery. The ex vivo organ perfusion systems provide unique opportunities to test the effectiveness of therapeutics on human organs before being used in patients. These treatments are invasive and technically demanding; thus, collaborations between nanoscientists and surgical teams are highly encouraged. The interdisciplinary approaches will transform research in nanotechnology, as well as translational research in organ transplantation.

## Local Delivery of Nanoparticle Therapies to Targeted Organs In Vivo—Back to Cancers

Recently, an in vivo lung perfusion system has been developed based on the success of EVLP. After removal of larger, detectable tumors, high-dose chemotherapy drugs are delivered only to the lung through this perfusion system to treat metastatic cells migrated from other organs into the lung, avoiding systemic side effects of chemotherapy drugs to other organs [40]. Nanoparticle-based anti-cancer therapies can be delivered using this system to further reduce the toxicity of chemotherapy to the lung, while evading the loss of nanoparticle dose to the liver, spleen, and kidney.

Of note, isolated limb infusion chemotherapy for melanoma [41] and isolated hepatic perfusion have been developed for cancer patients with liver metastases [42, 43]. These procedures are not without their risks; the protocols are complex and involve well-trained surgical teams and specialized equipment. However, as these systems successfully isolate delivery of drugs and thus evade the MPS, these systems represent a method of foundational exploration of nanoparticles and an immediate translational path to the clinic. These local delivery strategies, either in vivo or ex vivo, may also help to reduce the toxicity normally associated with the systemic delivery of nanoparticles [44]. It should be pointed out that many tumor cell metastases could affect multiple organs; active targeting is a better option in these conditions, especially when the metastatic tumors are too small to be detected yet.

In summary, the suggestions by Wilhelm et al. to recommit to foundational studies will no doubt lead to tremendous positive developments in the future. However, a step back is never taken with enthusiasm, and when there are more direct paths to translation through new technologies, it is paramount that we pursue those paths, translational and foundational, in parallel.

## Abbreviations

EPR: Enhanced permeability and retention
EVLP: Ex vivo lung perfusion
MPS: Mononuclear phagocytic system
